# Right ventricular strain as predictor of pulmonary complications in patients with femur fracture

**DOI:** 10.5830/CVJA-2017-011

**Published:** 2017

**Authors:** Hyun-Jin Kim, Hyung-Bok Park, Yongsung Suh, Yoon-Hyeong Cho, Tae-Young Choi, Eui-Seok Hwang,, Deok-Kyu Cho, Hyun-Jin Kim, Hyun-Sun Kim

**Affiliations:** Division of Cardiology, Department of Internal Medicine, Myongji Hospital, South Korea; Division of Cardiology, Department of Internal Medicine, Myongji Hospital, South Korea; Division of Cardiology, Department of Internal Medicine, Myongji Hospital, South Korea; Division of Cardiology, Department of Internal Medicine, Myongji Hospital, South Korea; Division of Cardiology, Department of Internal Medicine, Myongji Hospital, South Korea; Division of Cardiology, Department of Internal Medicine, Myongji Hospital, South Korea; Division of Cardiology, Department of Internal Medicine, Myongji Hospital, South Korea; Department of Translational Medicine, College of Medicine, Seoul National University, South Korea; College of Nursing and Research Institute of Nursing Science, Seoul National University, South Korea

**Keywords:** femur fracture, RV peak global longitudinal strain, pneumonia, pulmonary thromboembolism

## Abstract

**Background:**

Following femur fracture, medullary fat enters the systemic circulation and altered pulmonary haemodynamics may contribute to pulmonary complications. This study evaluated the association between right ventricular (RV) function and pulmonary complications in patients with femur fracture.

**Methods:**

Patients with a femur fracture who had undergone pre-operative echocardiography that included RV peak global longitudinal strain (RV GLS) were evaluated retrospectively between March 2015 and February 2016. Pulmonary complications were defined as the development of pneumonia or pulmonary thromboembolism during the first postoperative month.

**Results:**

Among 78 patients, pulmonary complications developed in eight (10.3%). The RV GLS value of all patients was lower than the normal range. In addition, the RV GLS value of patients with pulmonary complications was significantly lower than that of patients without pulmonary complications. Multivariate regression analyses found that worse RV GLS values independently predicted pulmonary complications [odds ratio (OR) 2.09, 95% confidence interval (CI) 1.047–4.151, p = 0.037]. Receiver operating characteristic curve analysis found that a RV GLS value of –14.85% was the best cut-off value to predict pulmonary complications; sensitivity: 75.0%; specificity: 62.9%. Moreover, patients with RV GLS values > –14.85% had significantly lower pulmonary complication-free survival.

**Conclusions:**

In patients with femur fracture, RV GLS values could help predict pulmonary complications. Therefore, patients with RV GLS values > –14.85 should be monitored closely before and after surgery for femur fracture.

## Introduction

Following femur fracture, a long-bone trauma, medullary fat enters the systemic circulation and altered pulmonary haemodynamics may contribute to pulmonary complications.^1^,^2^ Large amounts of medullary fat emboli entering the systemic circulation may produce multisystem dysfunction, more serious conditions, and pulmonary complications.^3^ In addition, following acute trauma, hormonal changes induce triglyceride hydrolysis and free fatty acid release, causing injury to the pulmonary capillary endothelium.^4^ All these changes after femur fracture, including altered pulmonary haemodynamics, hormonal changes and systemic inflammatory reactions, could worsen the clinical outcome of such patients. Accordingly, hospital stay or outcome can also be affected, not only by pulmonary complications with altered pulmonary haemodynamics but also from multisystem dysfunction.

Pulmonary vascular resistance (PVR) is an important component of pulmonary haemodynamics and a critical determinant of right ventricular (RV) systolic function.^5^ However, because PVR can only be measured directly by invasive right heart catheterisation, a non-invasive measurement of PVR is needed to evaluate acute trauma patients. Research has demonstrated that RV myocardial strain estimates RV function accurately and is correlated with the pulmonary haemodynamics of patients with pulmonary hypertension.^6^-^8^ Consequently, RV myocardial strain, as a measure of RV function, may provide new insights into detecting altered pulmonary haemodynamics and thereby predict pulmonary complications after femur fracture.

The aim of this study was to evaluate the association between pulmonary complications and RV function in patients with femur fracture.

## Methods

Data from 100 consecutive patients who visited a hospital for femur fracture between March 2015 and February 2016 and also underwent transthoracic echocardiography were retrospectively analysed. Among these, 22 patients were excluded from the study because the echocardiographic image quality was inadequate for quantitative analysis.

The study was approved by the institutional review boards of the hospitals, and was conducted according to the Declaration of Helsinki. Written informed consent was exempted by the institutional review boards.

Patients’ demographic and clinical characteristics were reviewed using electronic records. The following demographic and clinical characteristics were extracted: age, gender, height, weight, systolic blood pressure, diastolic blood pressure, current smoking status, and history of hypertension, diabetes, dyslipidaemia, coronary artery disease, and atrial fibrillation. Each patient’s body mass index [BMI (kg/m^2^)] was calculated. In addition, length of hospital stay was recorded.

The following laboratory data were extracted: haemoglobin, pro-brain-type natriuretic peptide, blood urea nitrogen, creatinine, estimated glomerular filtration rate, creatine kinase MB, troponin I, C-reactive protein, and D-dimer values. In addition, we recorded the dates of the diagnosis of pneumonia, pulmonary thromboembolism and all-cause death from the medical records.

Transthoracic echocardiographic images were reviewed for all patients. Transthoracic echocardiography was performed before the operation and images were obtained using the Vivid 7 or Vivid E9 echocardiography system (GE Vingmed, Horton, Norway). Left ventricular (LV) systolic function was assessed by calculation of LV ejection fraction (EF) using M-mode echocardiography.

RV systolic function was assessed from the following parameters: tricuspid annular plane systolic excursion (TAPSE), tissue Doppler-derived tricuspid lateral annular systolic velocity (RVs′), and RV fractional area change (FAC).^9^ RV FAC was calculated by tracing end-systolic and end-diastolic areas of the RV in apical four chamber views.^9^ Pulmonary artery systolic pressure was calculated from the maximal velocity of the tricuspid insufficiency jet and the estimated central venous pressure. Two-dimensional images of the apical four-chamber view were collected to analyse the longitudinal RV strain using a mean frame rate of 68 ± 10 frames per second. Analyses were performed using an off-line software program (EchoPAC PC version 113, GE Vingmed Ultrasound, Horton, Norway).

The endocardial border of the RV myocardium was delineated manually on an end-systolic frame, after which the software automatically drew the epicardial border. Manual adjustment was done for matching the actual borders of the regions of interest. Then the RV myocardium was traced frame by frame, and the longitudinal strain of the basal, middle and apical segments of the RV free wall and septum were obtained separately.

By averaging all segmental values, RV peak global longitudinal strain (RV GLS) was calculated using this software with two-dimensional speckle-tracking echocardiography.^10^ Because the longitudinal myocardial fibre length decreased during systole, the myocardial shortening was interpreted in negative values. Consequently, the more negative RV GLS values indicate improved or better strain.^11^ In patients with atrial fibrillation, all RV GLS measurements were averaged over three cardiac cycles.

The primary outcome was the development of a clinically apparent pulmonary complication during the one-month postoperative period. We defined a pulmonary complication as a composite outcome: development of either pneumonia or pulmonary embolism during the first postoperative month. Pulmonary embolism was confirmed by computed tomographic pulmonary angiogram.^12^ The diagnosis of pneumonia was based on a combination of physical signs and chest X-rays obtained by reviewing medical records.^13^

To assess the inter-observer variability of RV GLS, a second experienced independent investigator re-evaluated 20 randomly selected images using the same software. Intra-observer variability was evaluated: the first investigator who was blinded to the former results analysed RV GLS for each randomly selected image again at one month after the initial analysis.

## Statistical analsis

All categorical data are summarised as frequencies and percentages, whereas statistics for continuous variables are presented as means and standard deviations. The Pearson chi-squared test was used to compare categorical variables. The Student’s t-test was used to compare continuous variables and the Mann–Whitney U-test was used when the sample size of at least one group was less than 30.

Univariate analysis was followed by multivariate logistic regression analyses to evaluate potential risk factors for pulmonary complications with adjustment for other risk factors. Variables with a p-value less than 0.1 in univariate analysis were selected for inclusion in the multivariate regression model.

RV GLS was analysed as a continuous variable in univariate and multivariate models. We investigated the optimal cut-off value of RV GLS to predict pulmonary complications in patients with femur fracture using receiver operator characteristic (ROC) curve analyses. In addition, Kaplan–Meier survival analyses and log-rank tests were used to compare clinical event-free survival rates between groups stratified by the RV GLS cut-off value.

Then, to evaluate the consistency of results according to the identified cut-off value, RV GLS was analysed as a dichotomous variable in univariate Cox analysis. The inter- and intraobserver agreements were described by calculating the intra-class correlation coefficient (ICC). A p-value of less than 0.05 was considered statistically significant. All analyses were performed using SPSS 18.0 (SPSS Inc, Chicago, IL).

## Results

Seventy-eight patients with femur fracture were included; their mean age was 80.1 ± 9.1 years, and 59 (75.6 %) patients were female. Patients’ baseline characteristics are presented in Table 1. The mean hospital stay was 18.4 ± 7.8 days (median 17 days). All patients underwent successful surgery for femur fracture during hospitalisation.

**Table 1:** Baseline characteristics

*Clinical variables*	*All (n = 78)*	*Pulmonary complications (n = 8)*	*No complications (n = 70)*	*p-value*
Age, years	80.1 ± 9.1	83.4 ± 3.2	79.7 ± 9.5	0.310
Females, n (%)	59 (75.6)	5 (62.5)	54 (77.1)	0.395
SBP, mmHg	128.5 ± 20.6	126.5 ± 23.3	128.8 ± 20.5	0.728
DBP, mmHg	74.7 ± 11.9	71.9 ± 8.9	75.0 ± 12.3	0.488
Height, cm	156.0 ± 8.4	153.9 ± 5.5	156.2 ± 8.7	0.497
Weight, kg	54.7 ± 10.5	51.9 ± 7.6	55.1 ± 10.7	0.442
BMI, kg/m^2^	22.4 ± 3.6	21.9 ± 2.5	22.5 ± 3.7	0.767
Smoking, current, n (%	2 (2.6)	0 (0.0)	2 (2.9)	1.000
Hypertension, n (%)	59 (75.6)	7 (87.5)	52 (74.3)	0.671
Diabetes, n (%)	20 (25.6)	2 (25.0)	18 (25.7)	1.000
Dyslipidaemia, n (%)	9 (11.5)	0 (0.0)	9 (12.9)	0.586
Coronary artery disease, n (%)	7 (9.0)	1 (12.5)	6 (8.6)	0.546
Atrial fibrillation, n (%)	2 (2.6)	0 (0.0)	2 (2.9)	1.000
Hospital stay, days	18.4 ± 7.8	29.8 ± 17.0	17.1 ± 4.6	0.003

SBP, systolic blood pressure; DBP, diastolic blood pressure; BMI, body mass index.

Eight patients (10.3%) developed pulmonary complications during the first postoperative month. One patient (1.3%) developed pulmonary embolism and the other seven developed pneumonia. Among these, two patients (2.6%) died. The patients who developed pulmonary complications had significantly longer hospital stays than the patients who did not develop pulmonary complications (29.8 ± 17.0 vs 17.1 ± 4.6 days, p = 0.003).

Table 2 compares laboratory results and echocardiographic characteristics between the group with pulmonary complications and that with no complications. The patients in the group with pulmonary complications had significantly higher D-dimer values than the group with no complications (19 191.5 ± 16 257.0 vs 9 256.1 ± 10 304.5 ng/ml, p = 0.023). The groups did not differ significantly with regard to clinical characteristics, including previous medical history (hypertension, diabetes, dyslipidaemia, coronary artery disease and atrial fibrillation) (Table 1), and laboratory results, including cardiac enzymes, except for D-dimer values. In addition, echocardiographic characteristics were not significantly different between the two groups

**Table 2:** Laboratory tests and echocardiographic measurements

**	*All (n = 78)*	*Pulmonary complications (n = 8)*	*No complications (n = 70)*	*p-value*
Laboratory parameters				
Haemoglobin, g/dl	11.4 ± 1.8	10.3 ± 1.7	11.5 ± 1.8	0.091
Pro-BNP, pg/ml	1259.9 ± 4468.7	2375.3 ± 4237.1	1113.6 ± 4511.1	0.851
BUN, mg/dl	20.2 ± 9.0	22.7 ± 7.4	19.9 ± 9.1	0.205
Creatinine, mg/dl	1.1 ± 0.7	1.1 ± 0.2	1.1 ± 0.7	0.178
eGFR, ml/min/1.73 m^2^	60.1 ± 22.8	52.8 ± 8.3	60.9 ± 23.8	0.140
CKMB, ng/ml	2.0 ± 2.4	1.2 ± 1.0	2.1 ± 2.6	0.268
Troponin I, μg/l	0.1 ± 0.2	0.0 ± 0.1	0.1 ± 0.2	0.922
CRP, mg/dl	2.9 ± 5.0	5.6 ± 7.9	2.6 ± 4.6	0.161
D-dimer, ng/ml	10460.4 ± 11500.1	19191.5 ± 16257.0	9256.1 ± 10304.5	0.023
Echocardiographic parameters				
LVEF, %	61.6 ± 5.9	59.9 ± 9.9	61.8 ± 5.3	0.953
RV FAC, %	39.9 ± 8.2	37.0± 13.3	40.2 ± 7.5	0.442
RVs′, cm/s	14.3 ± 3.6	13.5 ± 3.7	14.4 ± 3.6	0.313
TAPSE, mm	18.5 ± 3.1	18.1 ± 2.1	18.5 ± 3.2	0.781
PASP, mmHg	36.0 ± 12.6	40.2 ± 17.7	35.4 ± 11.9	0.705

Pro-BNP, pro-brain-type natriuretic peptide; BUN, blood urea nitrogen; CKMB, creatine kinase MB; CRP, C-reactive protein; eGFR, estimated glomerular filtration rate; LVEF, left ventricular ejection fraction; RV, right ventricle; FAC, fractional area change; RVs′, tissue Doppler-derived tricuspid lateral annular systolic velocity; TAPSE, tricuspid annular plane systolic excursion; PASP, pulmonary artery systolic pressure.

Similarly, RV FAC, RVs′ and TAPSE values, known as measures of RV function, did not differ significantly between the two groups. Table 3 compares the RV strain values of the two groups. RV GLS values of all patients were lower than the normal range (approximately –28.0%.)^14^,^15^ RV GLS values of patients in the group with pulmonary complications were significantly lower than those of patients in the group with no complications (–12.40 ± 6.41 vs –17.14 ± 5.72 %, p = 0.036). With regard to segmental RV strain values, the apico-septal RV strain of patients in the group with pulmonary complications was noticeably worse than that in the group with no complications (–3.38 ± 12.98 vs –16.61 ± 11.04%, p = 0.010).

**Table 3:** RV strain analysis measurements according to pulmonary complications

**	*All (n = 78)*	*Pulmonary complications (n = 8)*	*No complications (n = 70)*	*p-value*
RV GLS, %	–16.66 ± 5.93	–12.40 ± 6.41	–17.14 ± 5.72	0.036
Basal septal	–15.35 ± 7.73	–14.63 ± 11.33	–15.43 ± 7.32	0.779
Mid-septal	–17.47 ± 6.94	–15.88 ± 10.91	–17.66 ± 6.43	0.662
Apico-septal	–15.26 ± 11.87	–3.38 ± 12.98	–16.61 ± 11.04	0.010
Basal lateral	–14.81 ± 14.19	–10.13 ± 11.47	–15.34 ± 14.44	0.166
Mid-lateral	–15.78 ± 10.66	–10.63 ± 13.18	–16.37 ± 10.28	0.121
Apico-lateral	–14.36 ± 13.38	–4.5 ± 18.87	–15.49 ± 12.31	0.108

RV GLS, right ventricular global longitudinal strain.

In univariate analyses (Table 4), worse RV GLS values were associated with pulmonary complications in patients [odds ratio (OR) 1.17, 95% confidence interval (CI) 1.007–1.369, p = 0.040]. Duration of hospital stay was also associated with pulmonary complications [OR 1.17, 95% CI: 1.041–1.307, p = 0.008]. Furthermore, in multivariate regression analyses, worse RV GLS values were independent predictors of pulmonary complications after adjustment for other relevant variables in patients [OR 2.09, 95% CI: 1.047–4.151, p = 0.037]. In addition, longer hospital stay was an independent predictor of pulmonary complications [OR 1.64, 95% CI: 1.053–2.560, p = 0.029].

**Fig. 1.**
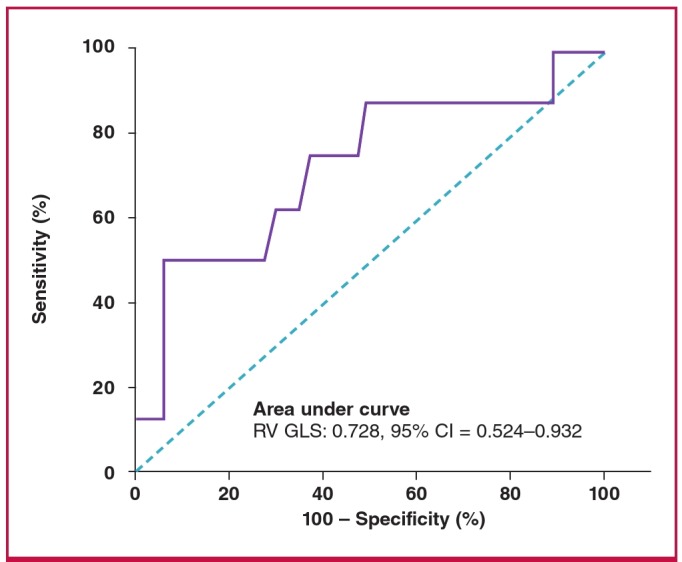
Receiver operating characteristic (ROC) curve analysis for the detection of pulmonary complications. The best cut-off value of RV GLS for the prediction of pulmonary complications was -14.85% (area under the curve: 0.728, p = 0.036). In patients with femur fracture, this value had a sensitivity of 75.0% and a specificity of 62.9% for correctly predicting pulmonary complications. CI, confidence interval, RV GLS, right ventricular global longitudinal strain.

**Fig. 2.**
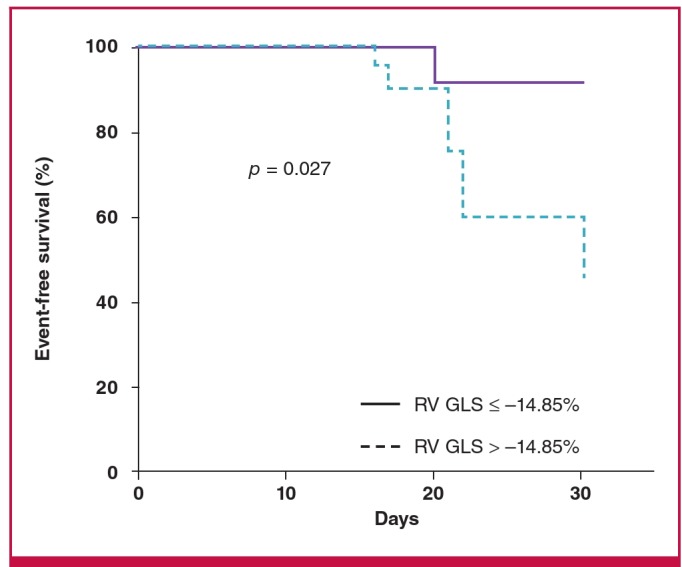
Clinical event-free survival curves based on Kaplan- Meier analysis. Patients were categorised into two groups: RV GLS > -14.85% and RV GLS ó -14.85%. The cumulative pulmonary complication-free survival rates of the two groups were compared using Kaplan- Meier survival curves and the log-rank test. Patients with RV GLS values > -14.85% had a significantly higher rate of pulmonary complications during the first postoperative month (p = 0.027). RV GLS, right ventricular global longitudinal strain.

ROC curve analysis identified RV GLS of –14.85% as the best cut-off value for predicting pulmonary complications; this value had a sensitivity of 75.0% and a specificity of 62.9% (Fig 1). Fig. 2 shows the cumulative clinical event-free survival rates of the two groups during the one-month postoperative period. The two groups were stratified by a RV GLS value of –14.85%. Patients with RV GLS values > –14.85% had a significantly higher rate of pulmonary complications during the first postoperative month than did patients whose RV GLS was ≤ –14.85% (log-rank test: p = 0.027). Based on univariate cox regression analysis, RV GLS values > –14.85% had borderline significance for the prediction of pulmonary complications [hazard ratio (HR) 7.60, 95% CI: 0.912–63.459, p = 0.061] during the one-month postoperative period (data are not shown).

The inter-observer agreement of RV GLS was excellent (ICC 0.987, 95% CI: 0.966–0.995, respectively). The degree of intra-observer agreement (ICC 0.989, 95% CI: 0.973–0.996) was similar to that of the inter-observer agreement.

**Table 4:** Univariate and multivariate analysis of RV GLS for predicting pulmonary complications

**	*Univariate*		*Multivariate*	
**	*OR*	*95% CI*	*OR*	*95% CI*
RV GLS, %	1.17	1.007–1.369	2.09	1.047–4.151
Hospital stay, days	1.17	1.041–1.307	1.64	1.053–2.560
D-dimer, ng/ml	1.00	1.00–1.00	1.00	1.000–1.000
Haemoglobin, g/dl	0.65	0.412–1.036	0.24	0.060–0.965

CI, confidence interval; OR, odds ratio.

## Discussion

Pneumonia and pulmonary embolism are important complications in older patients with femur fracture. Our findings indicate that pulmonary complications develop often, at a rate of 10.3%, in patients with femur fracture. Lower RV GLS values and longer hospital stay were good predictors for detecting pulmonary complications. In addition, our findings document that patients with impaired RV GLS values > –14.85% had significantly lower clinical event-free survival rates than patients with better RV GLS values.

After long-bone trauma, medullary fat enters the systemic circulation and fat emboli pass through the pulmonary capillaries, causing altered pulmonary haemodynamics and a systemic inflammatory reaction.^1^,^2^ Hormonal changes after trauma can also cause damage to the pulmonary capillary beds, causing altered pulmonary haemodynamics in animal models.^4^

Previous studies have demonstrated that when increased pulmonary vascular resistance is caused by vascular obstruction from fat emboli, RV afterload may increase and RVEF will decline.^16^-^18^ RV function reflects not only RV myocardial contractility but also the afterload effect of pulmonary vascular pathology.^19^ To our knowledge, no previous study has reported on the effects of femur fracture on pulmonary haemodynamics and RV function.

RV dysfunction has been shown to predict adverse clinical outcomes in patients with heart failure or myocardial infarction,^5^,^20^ therefore quantifying RV dysfunction would contribute to identifying at-risk patients, monitoring the effects of medical management and predicting clinical outcomes. However, it is difficult to assess RV function accurately using standard two-dimensional echocardiography imaging because the RV chamber has a complex shape.9K RV FAC and TAPSE have been established as echocardiographic parameters for assessing RV contractility or systolic function,^9^ and predicting adverse clinical events in patients with pulmonary embolism and myocardial infarction.^21^,^22^

Although the imaging quality has improved, delineation of the endocardial border for the measurement of RV FAC has variable reliability depending on the experience of the operator. In addition, velocity and displacement-based analyses, such as TAPSE, can be affected by tethering and cardiac translation and respiratory variation.^9^,^23^ Therefore, detecting subclinical RV dysfunction may be limited using conventional two-dimensional echocardiographic measurements. In our study, echocardiographic measures of RV function, including TAPSE and RV FAC, did not differ significantly with outcome; we did not detect differences in subclinical RV dysfunction between the group with pulmonary complications and that with no complications using conventional two-dimensional echocardiographic measures.

Two-dimensional strain echocardiography quantifies both regional and global myocardial function.^24^ Notably, detecting RV dysfunction using two-dimensional strain analysis can provide additional prognostic information and better predict outcomes than other traditional echocardiographic parameters in patients with myocardial infarction, pulmonary artery hypertension and heart failure.^24^,^25^ Because the RV muscle fibres are arranged longitudinally, most RV systolic function and RV stroke volume is generated by longitudinal shortening.^26^ Therefore, RV GLS is correlated with RV systolic function. A previous study demonstrated that RV GLS, including the interventricular septum and RV free wall, correlated significantly with RVEF based on cardiac magnetic resonance imaging.^27^

The reference value of RV GLS in normal subjects is about –28%,^14^,^15^ and the absolute values of RV GLS in our study patients were lower than the reference value. Relatively older age, altered pulmonary haemodynamics and decreased RV function due to acute trauma would tend to lower the absolute value of RV GLS.

A previous study revealed that RV GLS value ≥ –15.5% was associated with adverse clinical events and death in patients with inferior ST-segment elevation myocardial infarction (STEMI).^28^ The authors suggested that RV GLS was the major predictor of long-term clinical outcome in patients with acute inferior STEMI and preserved LVEF. Similarly, RV GLS value > –14.85% was associated with adverse clinical events in our study patients with preserved LVEF who had compromised pulmonary haemodynamics due to femur fracture.

It is notable from our analysis that the subclinical changes in RV function identified as decreases in RV longitudinal strain could be detected using two-dimensional strain analysis; these findings provide additional information to predict pulmonary complications in compromised pulmonary haemodynamics after acute long-bone trauma. Likewise, RV strain may help to further our understanding of pulmonary haemodynamic changes.

## Limitations

Some limitations of this study should be considered. First, this study was a retrospective observational study. Moreover, it was a single-centre experience with a relatively small sample size. Because quantitative analysis could have been affected by the quality of stored images, we excluded patients with inadequate echocardiographic image quality. Although a prospective study with more patients and a longer postoperative observation period may help identify additional factors, impaired RV GLS in patients after the acute trauma of long-bone fracture was significantly associated with more short-term clinical events and may provide useful information to manage trauma patients in real-world clinical situations.

## Conclusions

In patients with femur fracture, the short-term pulmonary complication rate was 10.3% and this was increased by worse RV GLS values and longer hospital stays. Because of the high incidence of pulmonary complications in femur fracture, patients with RV GLS values > –14.85% should be monitored closely before and after surgery to detect pulmonary events.
